# Rayleigh-wave attenuation across the conterminous United States in the microseism frequency band

**DOI:** 10.1038/s41598-021-89497-6

**Published:** 2021-05-12

**Authors:** Fabrizio Magrini, Lapo Boschi, Lucia Gualtieri, Vedran Lekić, Fabio Cammarano

**Affiliations:** 1 Department of Sciences, Università degli Studi Roma Tre, Rome , Italy; 2 Institute of Geosciences, Johannes Gutenberg University, Mainz, Germany; 3 Dipartimento di Geoscienze, Università degli Studi di Padova, Padua, Italy; 4 Sorbonne Université, CNRS, INSU, Institut des Sciences de la Terre de Paris, Paris, France; 5 Istituto Nazionale di Geofisica e Vulcanologia, Bologna, Italy; 6 Department of Geophysics, Stanford University, Stanford, CA USA; 7 Department of Geology, University of Maryland, College Park, MD USA

**Keywords:** Solid Earth sciences, Geophysics, Seismology

## Abstract

Mapping variations in the attenuation of seismic energy is important for understanding dissipative mechanisms in the lithosphere, and for modeling ground shaking associated with earthquakes. We cross-correlate ambient seismic signal recorded across the EarthScope Transportable Array in the 3–15 s period range. We apply to the resulting cross correlations a new method to estimate lateral variations in Rayleigh-wave attenuation, as a function of period, beneath North America. Between 3 and 6 s, our maps are dominated by a strong eastward decrease in attenuation. This pattern vanishes at longer periods, confirming early observations based on regional earthquakes. Attenuation maps and phase-velocity maps are anti-correlated at periods between 3 and 6 s, but the anti-correlation is also largely lost at longer periods. This corresponds to the attenuation coefficient decreasing with period more rapidly in the west than in the east, while the change in phase velocity with period is more uniform across the continent. Our results point to a transition in the properties of upper-crustal materials with depth, probably related to the closure of fluid-filled cracks and pores, and imply that measures of attenuation from seismic noise carry significant information on crustal rheology.

## Introduction

The crust is the most heterogeneous region of our planet, and its structure is the integrated result of magmatic, erosive, depositional, and tectonic processes over billions of years. Understanding its physical state and composition is essential for constraining the history of crustal production, destruction, and deformation. Seismic velocities have long been used to constrain crustal structure, but the non-uniqueness of their interpretation in terms of temperature, composition, density, and viscosity remains problematic. The amplitude of seismograms, on the other hand, is directly related to anelastic dissipation (including the effects of scattering); by quantifying such dissipation, one can attempt to constrain quantities that could not be extracted from seismic velocity alone, such as the abundance of water and partial melt^1–4^. Understanding how the mechanical properties of crustal rocks affect seismic amplitude also enhances predictions of earthquake-related ground motion, which is relevant for seismic hazard assessment and risk mitigation^5, 6^. This is crucial for large sedimentary basins, where the amplitude of ground oscillation has a strong impact on infrastructure safety.

Seismic surface waves are naturally sensitive to dissipation; it is known that, in addition to geometrical spreading, the amplitude of a surface wave decays with epicentral distance $\Delta$ according to the factor $\text{ e}^{-\alpha \Delta }$, where $\alpha$ is usually referred to as “attenuation coefficient”, or simply “attenuation”^7^. $\alpha$ changes with frequency (with lower-frequency waves sampling larger depths) and location. Dissipation is also often described by the quality factor *Q*, proportional to velocity and the inverse of $\alpha$; however, two equally valid definitions of surface-wave *Q* exist, one using group and the other phase velocity.

Existing estimates of $\alpha$ (or *Q*) carry large uncertainties. Global earthquake-based models^8, 9^ only afford limited lateral resolution, and regional higher-resolution models are restricted to seismically active areas. In principle, the cross correlation of seismic ambient noise^10, 11^ allows for enhancing resolution even in tectonically stable areas; but efforts to image attenuation based on seismic noise have been hindered by technical difficulties, related to the complex processing that this type of data requires^12^. Recent work by our team has contributed to resolving this issue^13, 14^, and should allow more robust estimates of attenuation as verified by a suite of numerical tests^15^. This study is the first systematic application of the new method to seismic ambient noise measured over a large continental area: the conterminous United States.

## Results

**Figure 1:**
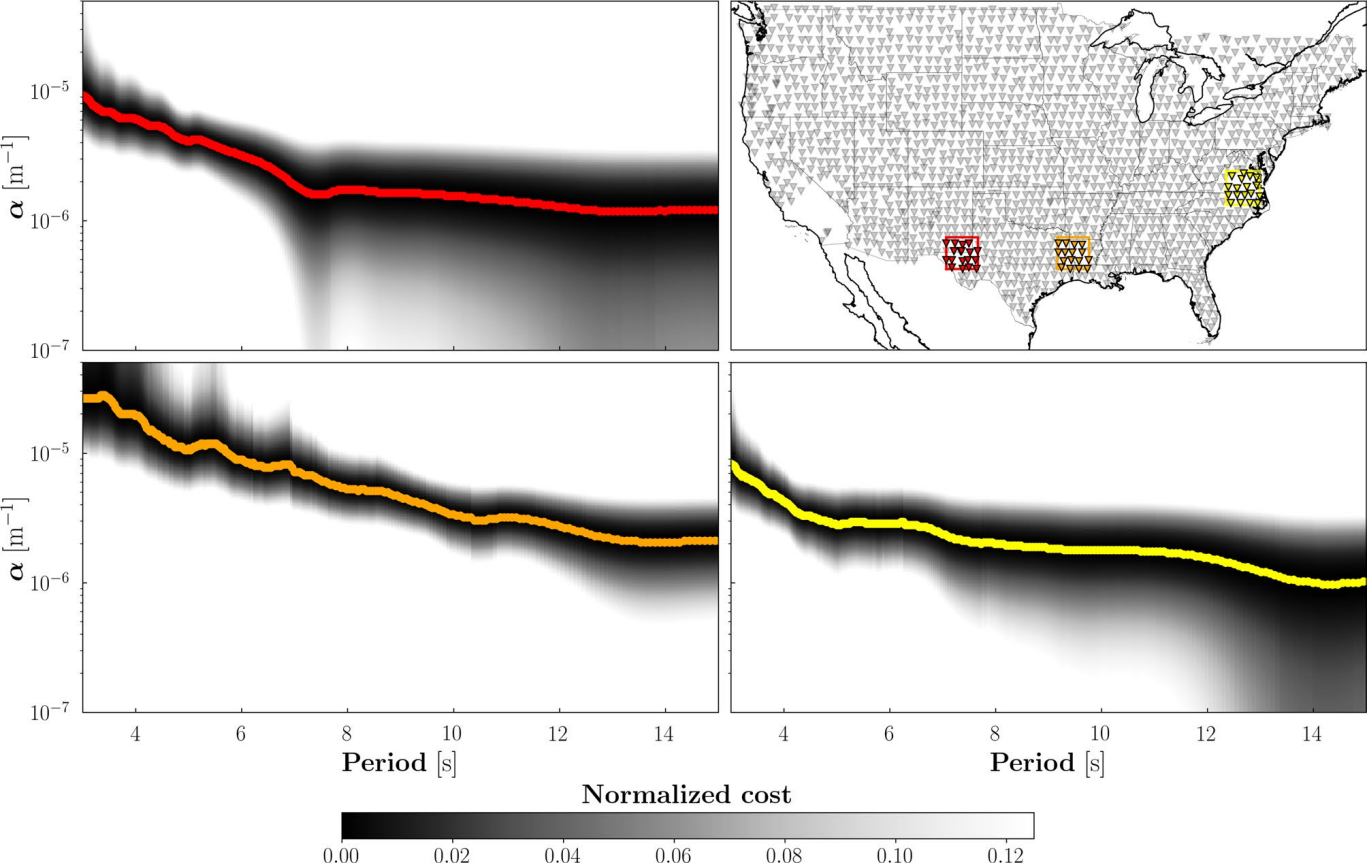
Map of the study area with three different blocks highlighted in red, orange, and yellow (upper right). The corresponding sub-arrays consist of 18, 16, and 18 receivers (colored triangles), respectively. All other seismic stations employed in these study are shown as gray triangles. The remaining subplots show the normalized cost (i.e., data misfit) as a function of period and $\alpha$, evaluated independently for each sub-array via grid search^15^. The three cost functions are characterized by well defined minima at each period (identified by the same colors previously associated to the blocks), which represent the final attenuation curves.

We used all the available seismic data from the transportable component of the USArray, consisting of over 400 broadband seismometers deployed in 1600 different locations across the United States and part of Canada. We subdivided the study area into relatively large overlapping (50%) blocks, with latitudinal extent of $2.5^{\circ }$, and longitudinal extent varying with latitude so as to keep the block area constant. Overall, this spatial parameterization allowed us to identify 440 overlapping sub-arrays (Fig. 1), each including five receivers at least (those of less than five receivers are discarded). For each sub-array, we first calculated inter-station Rayleigh-wave phase velocities (*c*), by cross correlation of continouos noise records in the frequency domain^16^. By nonlinear inversion^13–15^ of the data set thus compiled, we then retrieved 440 measurements (one per sub-array) of the frequency-dependent Rayleigh-wave attenuation coefficient $\alpha$ (Fig. 1). Our estimates of $\alpha$ cover the period range 3–15 s, sensitive to the shallow crust. So far, attempts to constrain the spatial variation of surface-wave attenuation in North America have been limited to periods of 8 s or longer^17–19^, and thus to larger depths.

### Phase-velocity and attenuation maps

**Figure 2:**
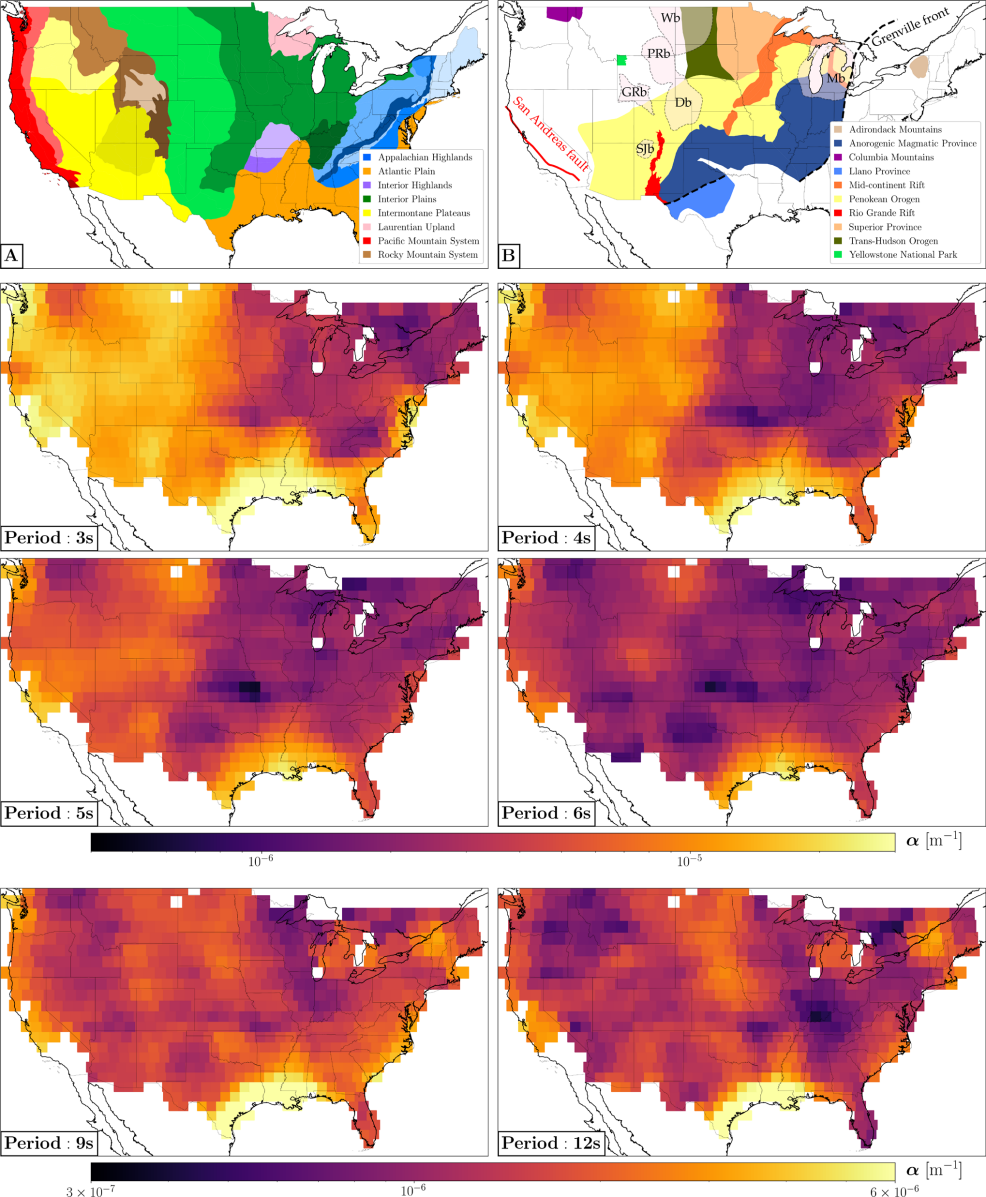
**(A)** Physiographic regions of the conterminous United States^22^; within each region, the boundaries of different provinces are highlighted by color shades. **(B)** Selected geological domains and tectonic lineaments. Transparent pink areas bounded by black dashed lines indicate sedimentary basins relevant to the discussion, i.e. Denver (Db), Greater Green River (GRb), Michigan (Mb), Powder River (PRb), San Juan (SJb), and Williston basin (Wb). The attenuation maps are presented with two different color scales: one for the period range 3–6 s, the other for periods $\ge 9 \, \hbox {s}$.

**Figure 3:**
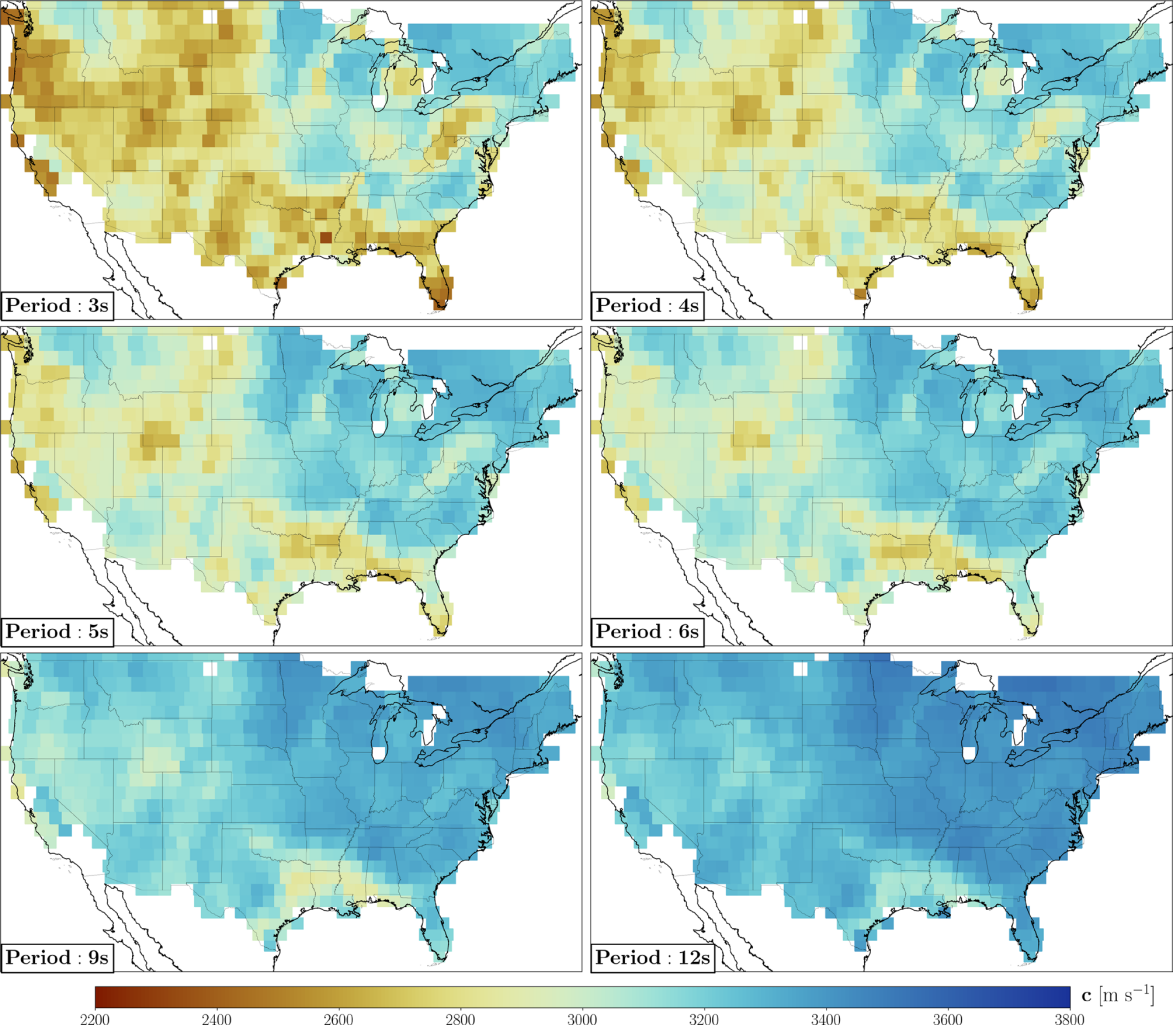
Rayleigh-wave phase-velocity maps of the study area at the periods of 3, 4, 5, 6, 9, and 12 s. The maps are highly correlated at all periods with those published in previous studies^20, 21^.

We translated our measurements of $\alpha$ into maps of seismic attenuation at different periods (Fig. 2), parameterized as pixel grids with $1^{\circ }$ spacing. The value shown in each pixel of Fig. 2 is the average of the estimates of $\alpha$ obtained from all sub-arrays sampling that pixel, weighted by the number of stations in the sub-arrays. The phase-velocity maps shown in Fig. 3 have been obtained analogously, from the Rayleigh-wave dispersion curves, and are highly correlated at all periods with those published in previous studies^20, 21^: averaged over frequency, the Pearson correlation coefficient between our phase-velocity maps and those of Ekström^21^ (after interpolating linearly their values in our grid) is $0.68 \pm 0.01$. Maps of the quality factor $Q = \frac{\pi f}{c \alpha }$ (*f* denotes frequency), obtained from the values of *c* and $\alpha$ shown in Figs. 2 and 3, are included in the supplementary information associated with this paper.

**Figure 4:**
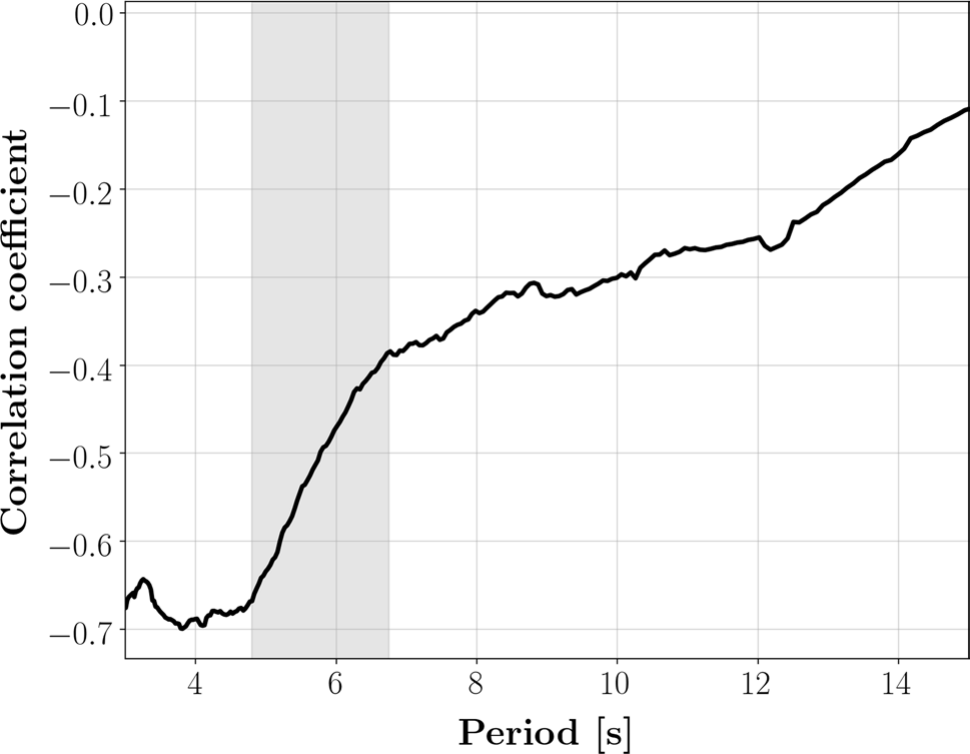
Pearson correlation coefficient as a function of period, obtained from the spatial correlation analysis of attenuation and phase-velocity maps. The area shaded in gray highlights a period range in which the correlation coefficient shows an abrupt increase.

At surface-wave periods between 3 and 5 s, the attenuation maps in Fig. 2 are characterized by strong lateral heterogeneity. Their most prominent feature is the large-scale trend of $\alpha$ decreasing eastward. Relatively high attenuation is observed in regions with thick sedimentary strata (e.g., Gulf of Mexico, Williston, Greater Green river, Michigan, San Juan) and regions undergoing active tectonics processes (e.g., San Andreas fault and most of the Pacific Mountain system). Relatively low attenuation characterizes the Columbia Mountains and most of the eastern, cratonic part of North America. A similar pattern is found in the phase-velocity maps of Fig. 3, and ascribed to lithologic variations across the conterminous United States^20, 21^. In fact, a spatial correlation analysis shows that our maps of attenuation and phase velocity are strongly anti-correlated in the period range 3-5 s, with values of Pearson correlation coefficient as large as 0.7 in absolute value (Fig. 4). In other words, we found that highly attenuating regions tend to be characterized by relatively low velocities, and vice-versa.

Our measurements at short periods (3–6 s) also show that Rayleigh-wave attenuation is strongly dependent on frequency, i.e. $\alpha$ quickly decreases with increasing period (Figs. 1 and 2). Since the frequency of a Rayleigh wave is strictly related to the depth range it samples (with lower-frequency waves sampling larger depths), this implies a rapid change of attenuation with depth. The derivative of $\alpha$ with respect to period is especially steep in the western part of North America (see, e.g., the fast decrease in attenuation characterizing the Intermontane Plateaus, Fig. 2); this translates into an abrupt decrease of the spatial anti-correlation between phase-velocity and attenuation maps in the period range $\sim$5–7 s (Fig. 4). This change in behavior exhibits itself geographically in the disappearance, at periods $\gtrsim 6 \, \hbox {s}$, of the large-scale pattern of attenuation decreasing eastward.

### Cluster analysis

**Figure 5:**
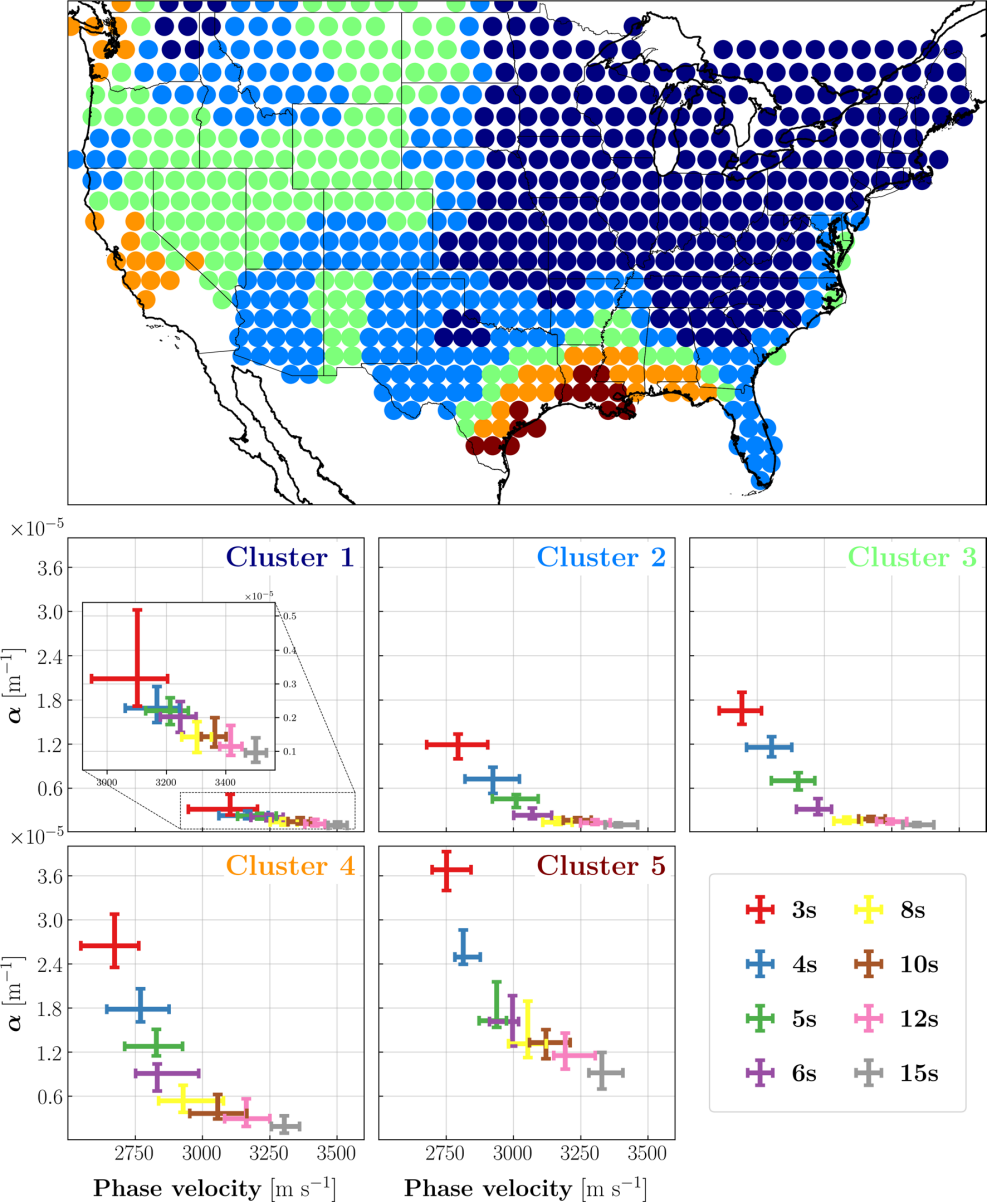
Results of cluster analysis, where each color identifies a different class. (Top) Spatial distribution of the five classes throughout the study area. (Bottom) Rayleigh-wave attenuation coefficient as a function of phase velocity. For each of the five clusters, 20th and 80th percentiles of $\alpha$ and *c* are shown in the form of error bars at different periods. At a given period, indicated by the color, the two bars intersect at the median value of $\alpha$ and *c*. Note the kink visible between 5 and 8 s in all sub-panels except for that relative to Cluster 1, corresponding with the eastern, cratonic North America. Because attenuation in that area is low, the values of Cluster 1 are zoomed to improve the visibility of $\alpha$ vs *c*.

To identify geographic patterns in surface-wave attenuation curves and relate them to various features of the Earth’s crust, we conducted a cluster analysis using the k-means algorithm^23^. After experimenting with different numbers of clusters, we partitioned the attenuation curves into five classes, whose spatial extents trace out coherent geographic patterns (Fig. 5). Despite the simplification inherent in describing the data space (i.e., the whole data set of attenuation profiles) in terms of five classes only, these appear to be well correlated with known geological provinces (see Figs. 2A,B and  5), indicating that attenuation bears important information on local geology. The clustering provided us with a quantitative regionalization of the study area, enabling us to identify geographic variations in the relationship between attenuation and phase velocity (Fig. 5, bottom panel). Specifically, we observe increasingly high attenuation from Cluster 1 (mostly associated with the eastern cratonic terrains) to Cluster 5 (corresponding with the Gulf coast sediments and the Mississippi embayment). Among the regions identified by these clusters, the most attenuating ones are also those associated with a faster decrease of $\alpha$ as a function of period. This result substantiates the observations (based on a visual inspection of the attenuation maps in Fig. 2) discussed above, i.e. that the frequency dependence of $\alpha$ is less pronounced in the cratonic part of North America. In addition, each of the identified regions presents a characteristic average trend of $\alpha$ vs *c*. This trend is characterized by a kink, which can be clearly identified between 5 and 8 s for all regions, except for Cluster 1.

## Discussion and conclusions

In the following, we discuss our findings in terms of three-dimensional crust and upper-mantle structure. It is understood that the maximum depth sampled by a surface wave at a given period grows approximately linearly with period itself^24^.

The observations enumerated above can be summarized as follows. (i) At periods $\lesssim 6 \, \hbox {s}$, Rayleigh waves in the western United States are characterized by relatively high attenuation $\alpha$ and relatively low phase velocity *c*; the opposite is true in the eastern United States (i.e., $\alpha$ and *c* heterogeneities are anti-correlated). (ii) In the same depth/period range, surface-wave attenuation drops quickly with increasing surface-wave period, especially in areas characterized by the presence of active tectonics and/or a thick sedimentary cover. (iii) A much lower anti-correlation between $\alpha$ and *c* heterogeneity is observed at periods between 6 and 15 s, where the large-scalelength east-west pattern is lost.

The absence of a strong large-scale pattern of attenuation decreasing eastward at periods $\gtrsim 6 \, \hbox {s}$ had already been observed in an early study based on a small number of regional earthquake measurements^25^. More recent studies that show a significant heterogeneity of attenuation in North America^2, 18^ are limited to even longer periods ($\ge 40 \, \hbox {s}$), outside the range considered here, and therefore to larger (uppermost-mantle) depths.

While temperature is generally believed to control attenuation at mantle depths^26–29^, temperature variations in our depth-range of interest are unlikely to result in significant attenuation heterogeneity; in the case of North America, this is inferred from the simple fact that the difference between crustal temperature in the east vs the west grows with depth^30, 31^, while the opposite is true of attenuation^25^. Mitchell^25^ also speculated that, at short surface-wave period, attenuation might be controlled by the density of fluid-filled fractures. Brittle, upper-crustal rocks of relatively low strength are traversed by a network of cracks and fractures, which in turn are filled by fluids; fluids are displaced by seismic waves, causing intrinsic absorption and, consequently, seismic attenuation^32–34^.

Our attenuation maps confirm this idea. Western north America is tectonically more active than eastern north America and, as a result, crustal materials in the western U.S. are characterized by a more pervasive distribution of fractures than in the east. We accordingly find higher values of $\alpha$ in the west, at the shortest periods investigated in this study (Figs. 2 and 3). The same mechanism that causes absorption and higher-than-average values of $\alpha$ also results in a reduction of the shear modulus^29^ (and therefore of surface-wave phase velocity, which is closely related to the shear modulus): this is confirmed by the mentioned anti-correlation of $\alpha$ and *c* heterogeneity (Fig. 4).

Increasing volumes of cracks and pores close under pressure with increasing depth^29^, resulting in the reduction of $\alpha$ with increasing period: we observe this most clearly in the western terrains and thick sedimentary basins (Fig. 5). The rapid decrease in the values of $\alpha$ in the west culminates (at periods $\gtrsim 6 \, \hbox {s}$) in the obliteration of the large-scale east-west dichotomy (Fig. 4), corresponding presumably to the nearly complete closure of fractures in most of the study area. At longer periods/depths, $\alpha$ and *c* continue to be anti-correlated albeit less significantly so.

Figure 5 is also in agreement with the idea that surface-wave attenuation be more sensitive than phase velocity to the presence of fluid-filled cracks and fractures^1–4^. In the 3-to-6 s period range, $\alpha$ drops with increasing period/depth more rapidly than *c* does, but this effect is reversed abruptly at longer periods (resulting in the kinks at about 6-8 s period in all regions of Fig. 5 except for the cratonic regions), i.e. after the closure of cracks.

The precise depth associated with the kinks in Fig. 5, or with the closure of most fractures, remains to be determined. Nonlinear inversions of surface-wave dispersion data are routinely conducted to estimate body-wave velocity structure^35, 36^, but their results carry significant uncertainty; $\alpha$ is less robustly constrained than dispersion, and therefore inverting our maps of $\alpha$ to determine anelastic structure at depth would carry even greater uncertainty. Accordingly, we have chosen to base our discussion on the more robust, although somewhat less informative, two-dimensional maps (Figs. 2 and 3) and frequency-dependent changes in the relationship between attenuation and phase velocity (Figs. 4 and 5). A recent study^37^ showed that shallow (1-3 km) changes in rock fabrics (including the closure of fractures) can significantly impact phase velocity and attenuation at surface-wave periods up to $\sim$10 s, characterized by much deeper sensitivity peaks. This means that, even if closing at a very shallow depth^38, 39^, fluid-filled fractures are still a valid explanation for our observations.

In summary, our interpretation of seismic attenuation in terms of the presence and closure of fluid-filled fractures in the upper crust is in agreement with both seismic data and regional tectonics. This supports the idea that, in general, fracture density affects seismic attenuation, and we infer that higher attenuation and lower velocities of surface waves might be observed in regions characterized by recent tectonic activity. Future work, perhaps accounting for independent rock and mineral physics data, is needed to further substantiate our speculations. Alternative explanations might also be considered, including e.g. the idea that attenuation could be controlled by the mechanical properties of a fractured/unconsolidated sedimentary cover overlying the crystalline basement, independent of the presence/absence of fluids. Scattering of seismic waves is also known to result in amplitude attenuation, but it is not expected to be great in large sedimentary basins, where, on the contrary, we systematically find large $\alpha$: we infer that attenuation is not dominated by scattering, at least in our frequency/depth range of interest. Finally, our observations can also be linked to studies of seismic anisotropy, which is also likely to be affected by fracture closing, and might therefore show a pattern of lateral variations similar to that of attenuation.

An additional, non-trivial implication of this study is that measures of surface-wave attenuation from seismic ambient signal carry significant information on crustal rheology, which cannot possibly be derived from seismic velocity data alone.

## Data and method

### PSD-normalized cross correlations

We exploited continuous, vertical-component seismograms recorded by the IRIS USArray Transportable Array (International Federation of Digital Seismograph Networks. https://doi.org/10.7914/SN/TA) between May 2004 and September 2019. Each seismogram has been demeaned, detrended, tapered ($5\%$), and bandpass filtered between 0.01 and 0.5 Hz before deconvolving with the instrument response to get displacement. To reduce the effects of temporal variability and/or seasonality of noise sources, we employed pairs of receivers that recorded simultaneously for at least 9 months. For a given pair of stations, the final cross-spectrum is obtained in the frequency domain by ensemble-averaging cross correlations calculated over 6-hour long windows, and normalizing by the average power spectral density (PSD) of the sub-array. Normalizing by the average PSD is beneficial to the subsequent processing for multiple reasons^13–15^. First, it allows one to relate the cross-correlation amplitude to ambient Rayleigh-wave attenuation, factoring out the parameters associated with frequency content and spatial distribution of the noise sources. Secondly, it mitigates the effect of anomalous signals such as large or nearby earthquakes. Finally, averaging over the PSDs computed individually for each sub-array helps minimize site effects inherent to a specific station or pair of stations.

The cross-spectra are processed following a three-steps procedure to better isolate the fundamental-mode amplitude used in the subsequent attenuation inversion^15^. In practice, we first inverse-Fourier transform the cross correlations; we then zero-pad the resulting signals in the time-domain at times corresponding to the velocity range $2\hbox {-}5 \, {\hbox {km\, s}}^{-1}$, so as to remove all signal that is much faster or slower than the typical fundamental-mode Rayleigh wave (i.e., Rayleigh-wave overtones and body waves); finally, we Fourier transform the padded cross correlations back to the frequency domain.

### Attenuation curves

We used the above cross-spectra to obtain robust measurements of attenuation, relying on the method described in^13–15^ and processing each sub-array independently. Given a sub-array, for each pair of receivers located at $\mathbf{x }_i$ and $\mathbf{x }_j$ we compute the inter-station phase velocity $c_{ij}$ by means of an automated algorithm^16^; this algorithm exploits, in the frequency domain, the zero crossings of the zeroth order Bessel function of the first kind $J_0$ associated with the considered frequency range and inter-station distance^40^. We then minimize the cost function^15^

1 $$\begin{aligned} C(\alpha , \omega )= \sum _{i,j} \vert \mathbf{x }_i-\mathbf{x }_j\vert ^2 \Biggr |\ \text{ env }\left[ \rho (\mathbf{x }_i, \mathbf{x }_j, \omega ) \right] - \text{ env }\left[ J_0\left( \frac{\omega \vert \mathbf{x }_i-\mathbf{x }_j\vert }{c_{ij}(\omega )}\right) \text{ e}^{-\alpha \vert \mathbf{x }_i-\mathbf{x }_j\vert } \right] \Biggr |^2, \end{aligned}$$

where $\vert \mathbf{x }_i-\mathbf{x }_j\vert ^2$ denotes inter-station distance, $\omega$ the angular frequency, and $\rho$ the PSD-normalized cross-spectrum. The envelope function env is implemented by fitting a combination of cubic splines^41^ to the maxima of the absolute value of the real part of their arguments^15^. The attenuation coefficient $\alpha$ is then retrieved by grid search over 275 different values distributed logarithmically between $5 \times 10^{-8}$ and $1 \times 10^{-4} \, {\hbox {m}}^{-1}$.

It is understood that, for each sub-array, all available cross correlations and dispersion curves are employed to minimize $C(\alpha , \omega )$. This contributes to “regularizing” the inversion (reducing unwanted effects like focusing/defocusing), thus allowing for more accurate estimates of $\alpha$^15^. The distribution of the number of station pairs per sub-array used to minimize $C(\alpha , \omega )$ is illustrated in Fig. S2. Earlier work by our team^13–15^ also indicates that, owing to the dense distribution of USArray stations and to the relatively long duration of the deployment, nonuniformity in the distribution of the noise sources is unlikely to affect much the estimates of attenuation derived with our method. The uncertainty on our attenuation measurements, evaluated by bootstrap analysis^15^, is discussed in thesupplementary information and shown in Fig. S3 in the form of maps of standard deviation.

### Clustering: k-means

The k-means algorithm allows for identifying the position of the centroids that best partition a data set into a predetermined number of clusters. These centroids represent data points in a (multi-) dimensional space, which can be used to classify data characterized by the same dimensionality based on different metrics. Here, we used the Euclidean distance and the belonging class of each measurement is determined by the closest centroid. The data set fed to the k-means algorithm consisted of a $695 \times 254$ matrix, where 695 is the number of pixels in our maps of Figs. 2 and 3, and 254 are the values of $\alpha$ per attenuation curve (i.e., the dimensionality of the data set).

## Supplementary Information

Supplementary Information.
