# Supplementary material for: Rayleigh-wave attenuation across the conterminous United States in the microseism frequency band
Source: Sci Rep. 2021 May 12;11:10149. doi: 10.1038/s41598-021-89497-6 (PMC8115131; doi:10.1038/s41598-021-89497-6)
Supplement: Supplementary file 1 — Supplementary Information. [file 41598_2021_89497_MOESM1_ESM.pdf]

Rayleigh-wave attenuation across the conterminous United States in the microseism frequency band – Supplementary information

Fabrizio Magrini[1,2], Lapo Boschi[3,4,5], Lucia Gualtieri[6], Vedran Lekić[7], and Fabio Cammarano[1]

[1]Department of Sciences, Università degli Studi Roma Tre, Italy
[2]Institute of Geosciences, Johannes Gutenberg University, Mainz, Germany
[3]Dipartimento di Geoscienze, Università degli Studi di Padova, Italy
[4]Sorbonne Université, CNRS, INSU, Institut des Sciences de la Terre de Paris, France
[5]Istituto Nazionale di Geofisica e Vulcanologia, Bologna, Italy
[6]Department of Geophysics, Stanford University, Stanford, CA, USA
[7]Department of Geology, University of Maryland, MD, USA

April 20, 2021

Uncertainty of the attenuation maps

Similar to *Magrini and Boschi* (2020), we evaluated the uncertainty of our attenuation maps via bootstrap analysis. In practice, we performed 100 inversions (by minimization of the cost function in eq. (1)) for each of the sub-arrays described in the main text, removing 15% of the available station pairs at each iteration. This allowed us to calculate 100 attenuation curves, that we used to produce maps (parameterized in the same fashion of Figs. 2 and 3) of the standard deviation. The results of this analysis are illustrated in Fig. S3; they show that, in most of the study area and at all investigated periods, the standard deviations we obtain are small compared to the amplitude of spatial variations of α discussed in the main text (Fig. 2). This analysis supports the robustness of the lateral variations visible in our attenuation maps, and the general validity of our interpretation.

Robustness of the five clusters

We evaluated the robustness of the clusters presented in the main text by performing a silhouette analysis (Fig. S4) (*Rousseeuw*, 1987). The silhouette analysis provides a measure of how similar a data point is to the cluster to which it belongs, as opposed to the other clusters; this is quantified by the silhouette coefficient (henceforth SC), which is a rational number falling on the closed interval $[-1, 1]$. The goodness of the classification for each given sample increases with increasing SC, and an SC equal to zero indicates that the sample lies on the boundary separating the belonging cluster from the neighbor one. Fig. S4 shows that

the average SC associated with the five clusters discussed in the main text is relatively high (0.53), and can therefore be considered robust.

References

Aki, K., and P. G. Richards, *Quantitative seismology*, 2002.

Magrini, F., and L. Boschi, Surface-wave attenuation from seismic ambient noise: numerical validation and application, *Journal of Geophysical Research: Solid Earth*, *126*, doi:10.1029/2020JB019865, 2020.

Romanowicz, B., Inversion of surface waves: a review, *International Geophysics Series*, *81*(A), 149–174, 2002.

Rousseeuw, P. J., Silhouettes: a graphical aid to the interpretation and validation of cluster analysis, *Journal of computational and applied mathematics*, *20*, 53–65, 1987.

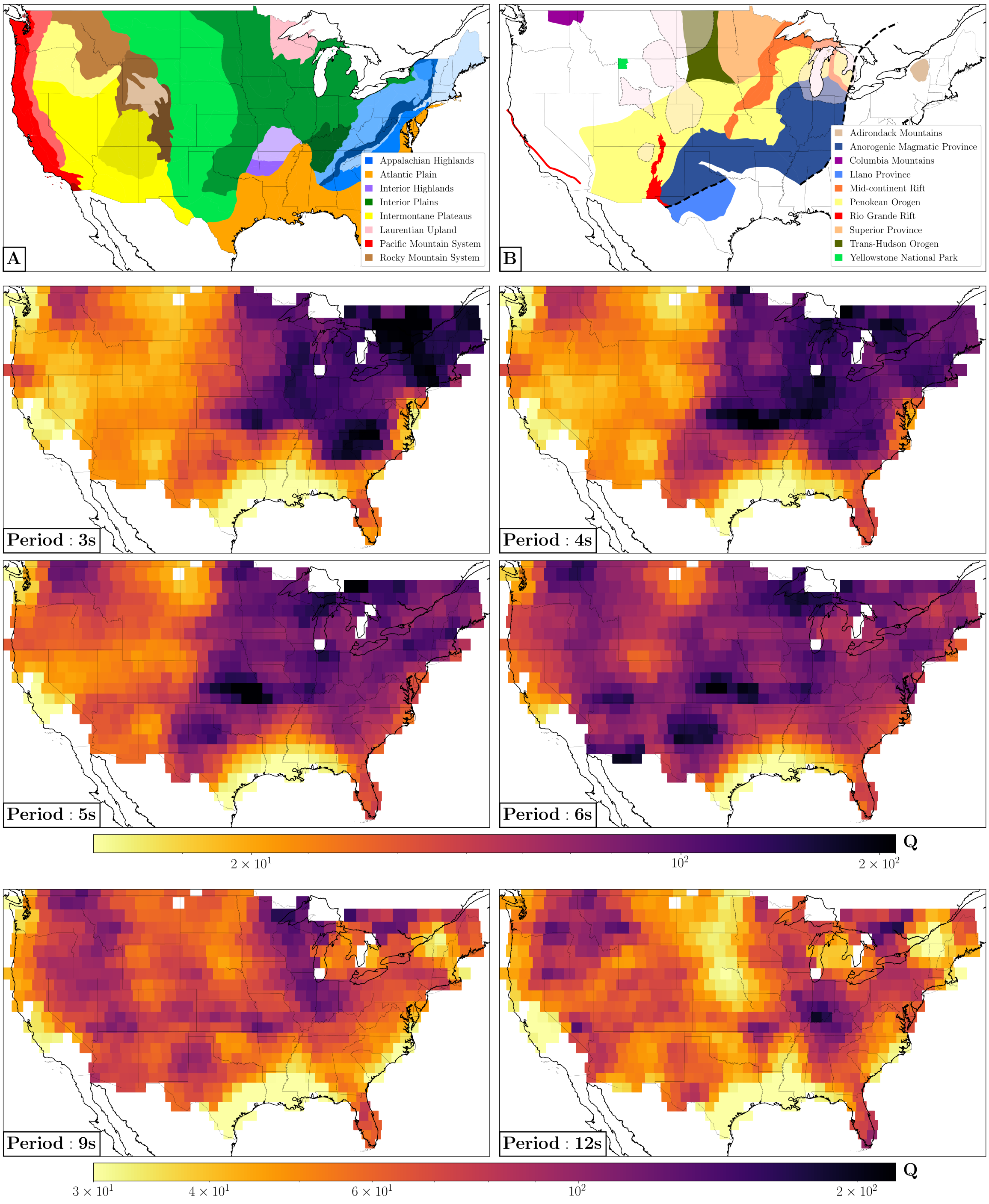

Figure S1: Same as Fig. 2 of the main text, but referred to the lateral variations of the quality factor Q. In each pixel, the values of Q have been derived from our maps of α and c through to the relation $Q = \frac{\pi f}{c\alpha}$ (e.g., *Romanowicz*, 2002), where f denotes frequency. Care should be taken when comparing estimates of Q from different surface-wave studies, because the definition of surface-wave Q is inherently ambiguous: see e.g. *Aki and Richards* (2002), sec. 7.3.4 and eq. (7.93).

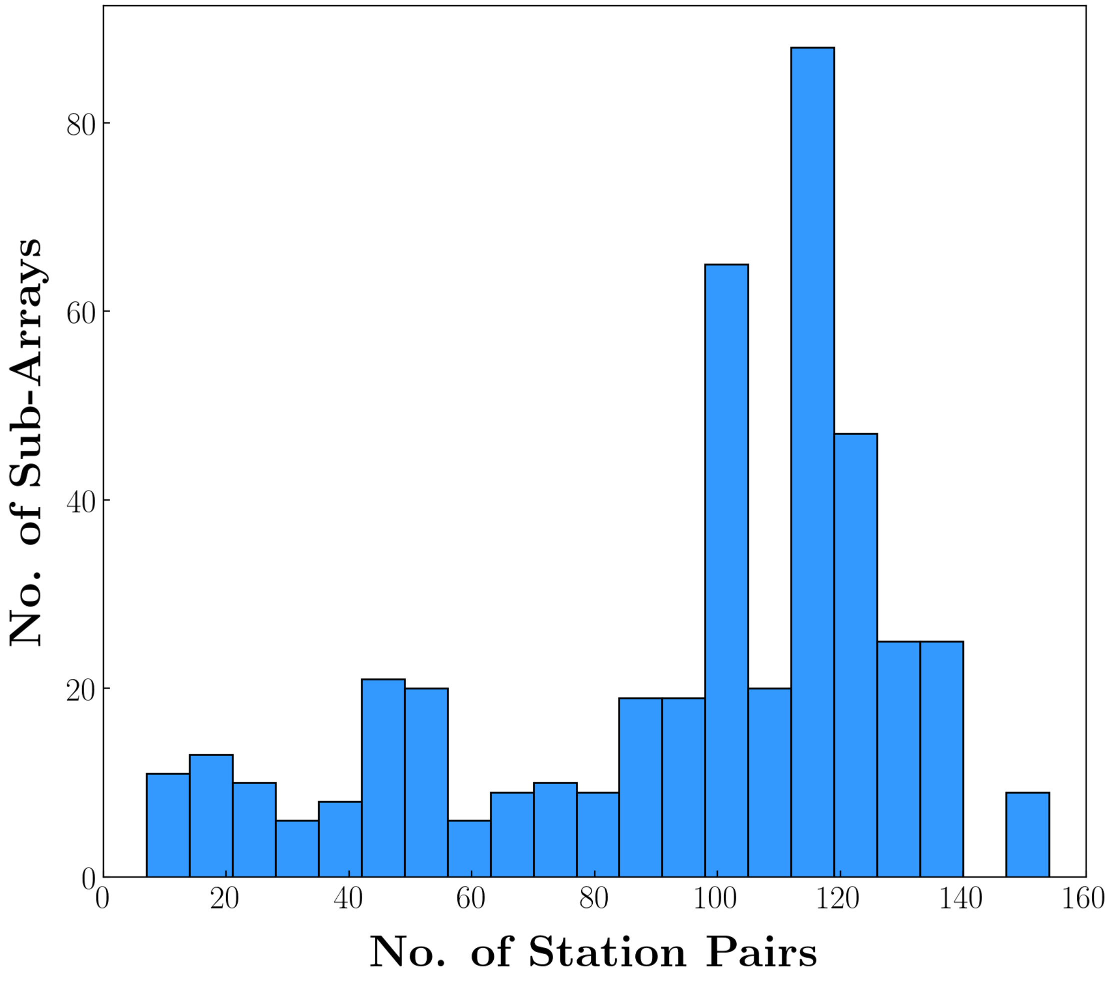

Figure S2: Distribution of number of station pairs in the sub-arrays used to obtain local attenuation measurements. The mean and median of the distribution are 94.2, 104.0, respectively.

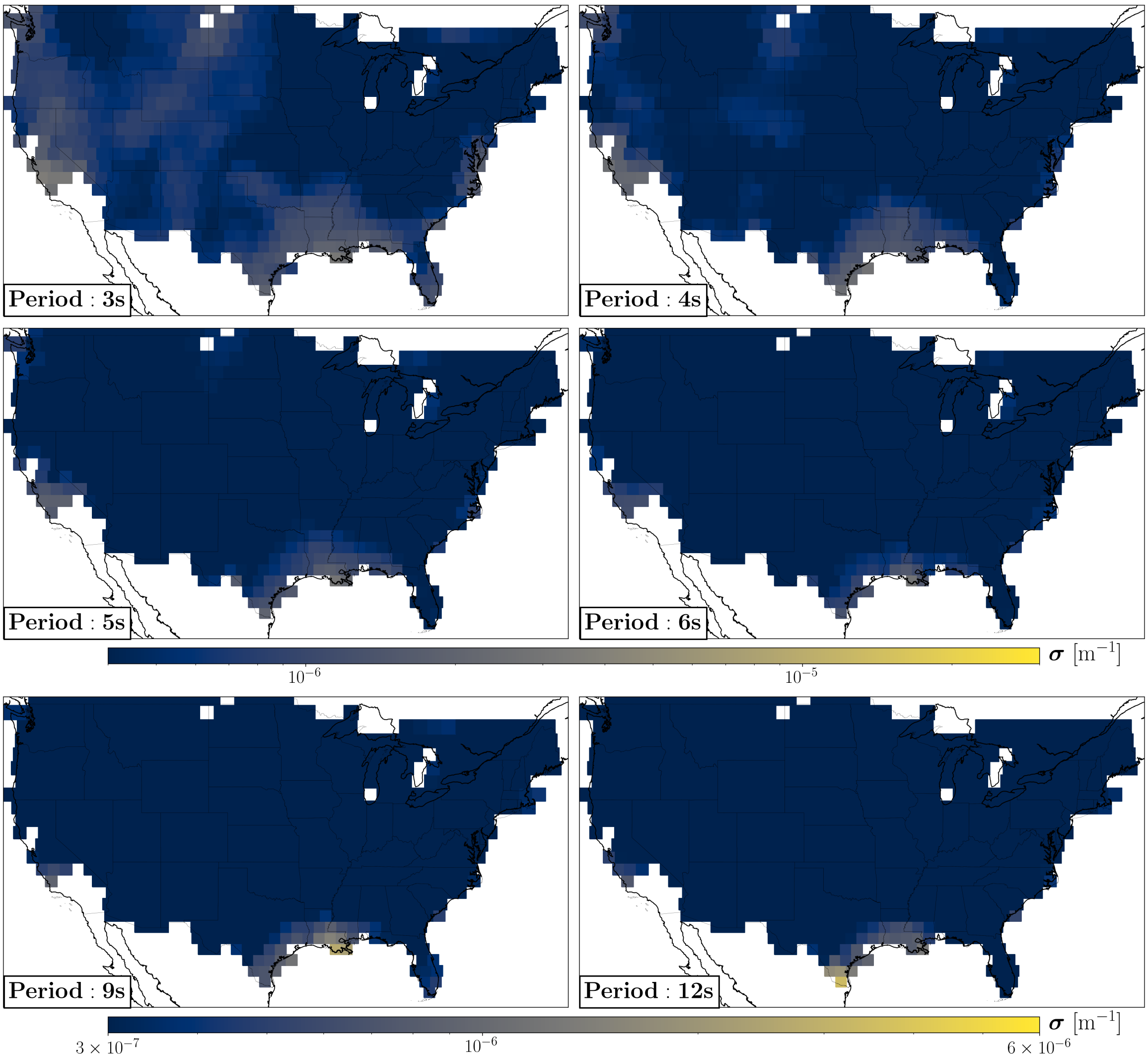

Figure S3: Maps of the standard deviation σ obtained on our attenuation measurements via bootstrap analysis. The maps are presented with two different color scales: one for the period range 3-6 s, the other for periods ≥ 9 s. These color scales correspond to those employed in Fig. 2 of the main text. Note that the standard deviations are much smaller than the spatial variations shown in Fig. 2 across most of the study area at all periods.

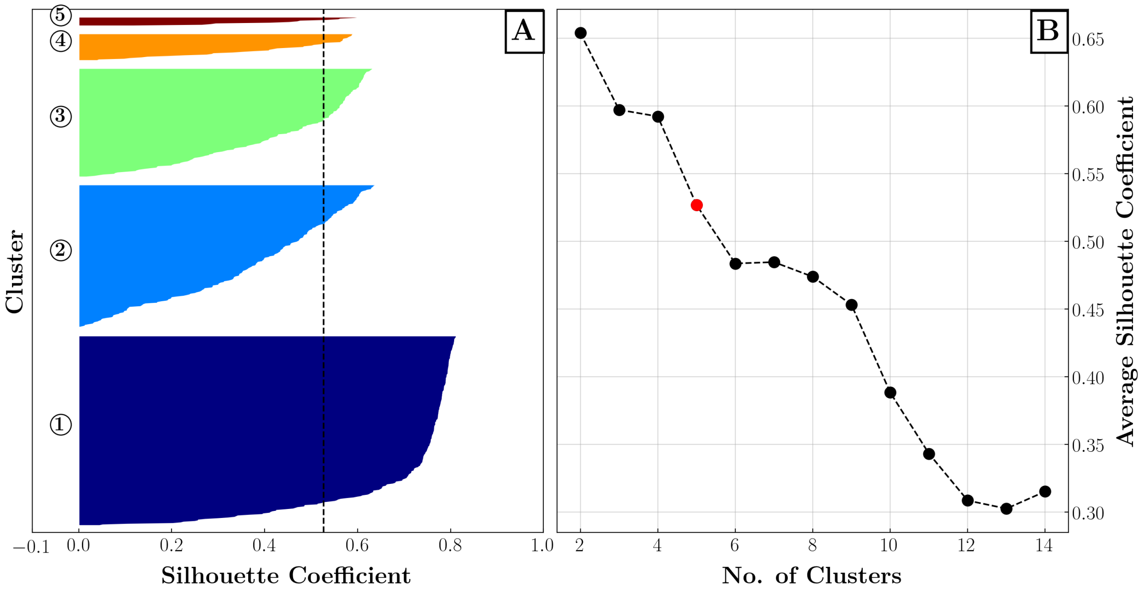

Figure S4: Results of the silhouette analysis performed to evaluate the robustness of the five clusters. (a) SC obtained for each sample belonging to the five classes; for each cluster, the value of SC of each sample is indicated by a horizontal line starting from zero. The black dashed line denotes the average SC (0.53). (b) Average SC as a function of the number of clusters; the red dot indicates the number of clusters used in this study (five).
